# Brain–kidney crosstalk

**DOI:** 10.1186/cc13907

**Published:** 2014-06-05

**Authors:** Arkom Nongnuch, Kwanpeemai Panorchan, Andrew Davenport

**Affiliations:** 1 Renal Unit, Department of Medicine, Faculty of Medicine, Ramathibodi Hospital, Mahidol University, 34/1 Soi Saengsuksa School, Isarapap Road, Min Buri, Bangkok 10510, Thailand; 2 UCL Centre for Nephrology, Royal Free Hospital, University College London Medical School, Rowland Hill Street, London NW3 2PF, UK; 3 Bumrungrad International Hospital, 33 Sukhumvit Soi 3, Wattana, Bangkok 10110, Thailand

## Abstract

Encephalopathy and altered higher mental functions are common clinical complications of acute kidney injury. Although sepsis is a major triggering factor, acute kidney injury predisposes to confusion by causing generalised inflammation, leading to increased permeability of the blood–brain barrier, exacerbated by hyperosmolarity and metabolic acidosis due to the retention of products of nitrogen metabolism potentially resulting in increased brain water content. Downregulation of cell membrane transporters predisposes to alterations in neurotransmitter secretion and uptake, coupled with drug accumulation increasing the risk of encephalopathy. On the other hand, acute brain injury can induce a variety of changes in renal function ranging from altered function and electrolyte imbalances to inflammatory changes in brain death kidney donors.

## Review

Acute kidney injury (AKI) is a worldwide problem, associated with increasing morbidity and mortality, and can be considered as a systemic inflammatory condition that may have substantial effects on various distant organs [1-3].

Patients with AKI commonly develop confusion, which may be present at the onset of AKI or develop subsequently. The cause of neurological disturbance is often multifactorial, as the majority of patients who develop AKI do so on a background of small vessel disease associated with cardiac failure, diabetes and hypertension – resulting in a spectrum of fluctuating clinical signs ranging from headache, visual abnormalities, tremor and asterixis through to multifocal myoclonus, chorea, seizures and coma. As such, many older patients who develop AKI may already have established small vessel ischaemic brain disease characterised by white matter changes, lacunar infarcts and disturbed cerebral autoregulation, so predisposing them to additional cerebral injury. Moreover, neurological problems in AKI may not only be directly related to uraemic toxin accumulation, but may also develop due to electrolyte imbalance, drug toxicity, thiamine deficiency, and also additionally the effects of renal replacement therapy, including dialysis disequilibrium [4]. Encephalopathy may also occur in the setting of sepsis, as sepsis is not only the commonest precipitating factor for hospital-acquired AKI but is also more likely to develop in patients with AKI. Because AKI is an inflammatory condition, platelet activation and increased thrombin generation increase the risk of cerebral ischaemia due to cerebral capillary thrombosis typically found in cases of malaria-induced and leptospirosis-induced AKI. In addition, as drug pharmacokinetics may be altered in AKI, the risk of drug-induced encephalopathy is increased.

On the other hand, acute brain injury can lead to neurohumeral changes that directly affect the kidney by increasing renal sympathetic nervous system activity, so altering renal blood flow and glomerular filtration, and by altering vasopressin release lead to changes in sodium and water balance. In addition, acute brain injury is also a cause of cerebral salt wasting, and also severe brain injury leading to brain death results in haemodynamic instability, hormonal disturbance and increased immunologic response, triggering an inflammatory cascade in several organs, including the kidney. Clinically, studies have reported a link between brain death and increased inflammatory response in the kidneys used for organ donation, leading to donor graft dysfunction [5,6]. Similarly, renal dysfunction in the setting of both acute ischaemic and haemorrhagic stroke is associated with increased hospital stay and mortality [7].

Although there is overlap between encephalopathy in patients with AKI and those with end-stage chronic kidney disease (CKD), this review will concentrate mainly on the effects of AKI on the brain, and conversely the effects of acute brain injury on the kidney, rather than discussing the effects of CKD on cerebral function or the insults that affect the brain and kidney.

## Acute kidney injury and the brain

**Figure 1:**
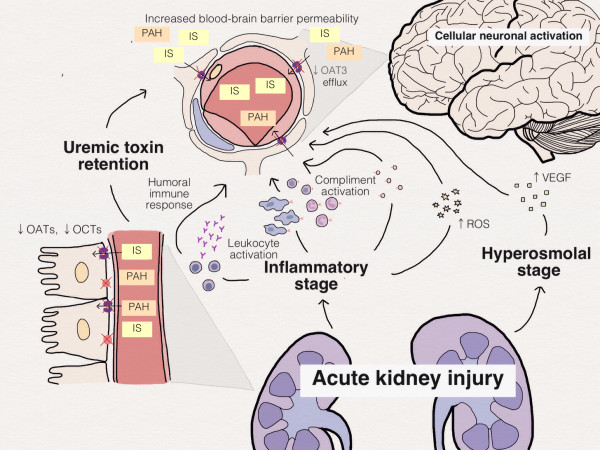
**Schematic diagram depicting the effect of acute kidney injury on integrity of the blood–brain barrier.** IS, indoxyl sulphate; OAT, organic anion transporter; OCT, organic cation transporter; PAH, para-aminohippuric acid; ROS, reactive oxygen species; VEGF, vascular endothelial growth factor.

**Figure 2:**
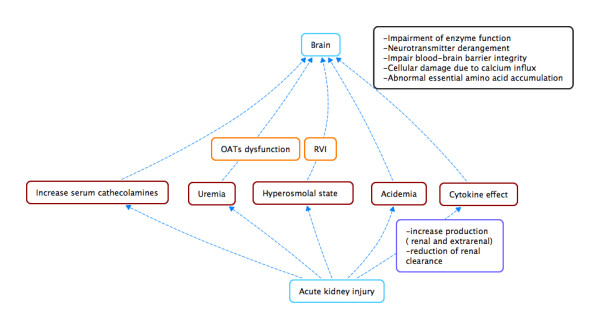
**Schematic cartoon showing potential pathways of brain and kidney crosstalk.** OAT, organic anion transporter; RVI, regulatory volume increase.

Injury in one organ can lead to changes in a distant organ, and *vice versa*. The interactions between the liver and the kidney [8] and between the heart and the kidney are the best categorised and often referred to as the hepato-renal syndrome and the cardio-renal syndromes [9]. The kidney not only plays a key role in maintaining electrolyte, acid–base, sodium and water homeostasis, but also in metabolising hormones and excreting toxins. As such, AKI is likely to challenge cerebral homeostasis both at the cellular level [10] and also by altering neurotransmitter concentrations, circulating cytokines, acid–base imbalance, haemostasis, and drug metabolism (Table 1 and Figures 1 and 2).

**Table 1:** Summary of mechanisms associated with cerebral dysfunction during acute kidney injury

**Mechanism**	**Results**
Impaired blood–brain barrier integrity	Alteration of essential amino acid concentrations, inflammatory mediators and organic osmolyte in the brain
Neurotransmitter derangement	Decreased cerebral norepinephrine, epinephrine and dopamine may lead to impaired locomotor activity
Trigger inflammatory cascade	Three waves of danger signalling unleashing uric acid, Weibel–Palade bodies and high mobility group box 1 protein
Acid–base disturbance	Activation of acid-sensing ion channels leading to cellular injury
	Local vasodilatory effects as a result of cerebral oedema
Organic osmolyte and brain water disturbance	Increased intracellular idiogenic osmoles and brain water
Alteration of drug pharmacokinetics	Downregulation of organic acid transporters and organic cation transporters
	Alteration of protein binding of drug
	Impaired renal and hepatic clearance of drug

These potentially pathophysiological changes contribute to both direct and indirect insults to the brain. For example, there is an increased risk of both cerebral haemorrhage and thrombosis with AKI due to the combination of endothelial injury, abnormal platelet function and coagulation cascade dysregulation – particularly associated with cerebral involvement with Dengue fever and other haemorrhagic viral infections, leptospirosis and both thrombotic thrombocytopenic purpura and haemolytic uraemic syndrome [4]. Furthermore, in the setting of AKI secondary to Goodpasture’s syndrome or systemic vasculitides patients may develop steroid-induced psychosis, on the one hand, and the risk of overimmunosuppression in the immunocompromised patient, on the other, including AKI in the renal transplant recipient increasing the risk of encephalopathy due to primary brain infections with bacterial, viral, fungal or atypical organisms. In addition, monoclonal and polyclonal antibodies to lymphocytes may precipitate a sudden cytokine release resulting in encephalopathy or aseptic meningitis, and excess immunophyllin dosing may also cause an encephalopathy associated with characteristic cerebral white matter changes.

## Blood–brain barrier integrity and disruption in acute kidney injury

The blood–brain barrier (BBB) and the blood–cerebrospinal fluid barrier (BCSFB) are designed to maintain cerebral homeostasis. These structures play a major role in regulating the transport of amino acids, proteins, and essential nutrients into and out of the brain. The integrity of the BBB and the BCSFB depends on the maintenance of tight junctions between cerebral endothelial cells and the choroid plexus, supported by astrocytes, which are specialised glial cells. Disruption of the BBB and the BCSFB can allow the influx of proteins, amino acids and inflammatory cells into the brain tissue.

The effects of AKI on BBB permeability are not well characterised. However, there are numerous studies reporting disruption of BBB integrity in various inflammatory models including sepsis [11,12], liver failure [13,14], and neurological disease [15,16]. AKI is recognised to lead to systemic inflammation, due to the inflammatory response within the kidney [17,18], so AKI may similarly share the common inflammatory pathways that increase endothelial permeability [12] and BBB permeability. AKI increases proinflammatory cytokine generation, but also reduces cytokine clearance – so increasing the overall inflammatory response [19,20], leading to leukocyte activation and recruitment, activation of the complement cascade pathway, and amplifying the humoral immune response [21]. A sudden reduction in kidney function leads to toxin accumulation and increased serum osmolality, which can directly stimulate vascular endothelial growth factor [22] as well as increasing reactive oxygen species, resulting in endothelial injury [22,23], BBB disruption, upregulation of toll-like receptors [24], neuronal activating protein [10,25] and brain transporters (Figure 1).

Animal models of AKI have demonstrated disruption of the BBB with extravasation of albumin-bound Evans blue dye compared with sham-operated controls [12,25]. Models of local inflammation induced by tumour necrosis factor alpha or generalised sepsis cause activation of brain astrocytes, resulting in changes to the BBB, and increased permeability. These animal studies support the concept that inflammation associated with AKI with increased circulating cytokines leads to disruption of the BBB, allowing increased access to inflammatory cells, cytokines, complement, amino acids and organic osmolytes.

## Changes in cerebral neurotransmitters in acute kidney injury

Complex brain function is tightly controlled by multiple interneuronal synapses using combinations of excitatory and inhibitory neurotransmitters. Derangements in the metabolism of these neurotransmitters may variably disrupt cerebral function, ranging from coma to hyperexcitation states. Interactions between the kidney and the brain in terms of changes in cerebral neurotransmitters have been well described in animal models for more than three decades [10,26].

Transport across the BBB is normally highly regulated. Most animal studies in AKI have centred on amino acid transport; for example, the Na^+^-independent cationic amino acid transporter 1 (CAT1/SLC7A1), which regulates l-arginine influx into the brain [27]. As l-arginine is the precursor for guanidine compounds, including taurine, alanine, glycine, guanidinoacetic acid and creatine, any interference with this transporter during AKI may both potentially cause the accumulation of and also the depletion of amino acids and neurotransmitters within the brain. Some amino acids are strictly regulated to have very low concentrations in brain tissue by active efflux into the circulation via a series of transporters. Glutamate, aspartic acid, glycine, gamma aminobutyric acid, and taurine are actively exported out through excitatory amino acid transporters, so disruption of these transporters due to changes in BBB integrity in AKI can lead to accumulation within the brain.

Brain catecholamine concentrations are often depleted in animal models of AKI [28]. Changes in catecholamine neurotransmitter metabolism have been associated with impaired motor activity [29], which is supported by experimental studies reporting a fall in plasma valine and threonine but increased brain phenylalanine, tyrosine, and histidine [30], so potentially increasing the amount of cerebral monoamine neurotransmitters, with alteration of mental status and motor disability [31] (Table 2).

**Table 2:** Summary of animal studies of different models of acute kidney injury reporting effects on the brain

**Study**	**Subjects**	**Model**	**Findings**
Ali and colleagues [26]	Rats	Nephrectomy	Increased plasma norepinephrine, epinephrine and dopamine
			Decreased cerebral norepinephrine, epinephrine and dopamine
Jeppsson and colleagues [30]	Rats	IRI	Reduced plasma valine and threonine but increased plasma phenylalanine
			Increased phenylalanine, tyrosine, and histidine, but decreased threonine in the brain
Adachi and colleagues [29]	Rats	IRI	Unchanged in cerebral norepinephrine and serotonin turnover as well as brain water content
			Decrease in cerebral dopamine metabolism and motor activity
Palkovits and colleagues [10]	Rats	Nephrectomy, drugs induce AKI	Moderate increase in neuronal activation in the biogenic amine expressing cell group
			Strongly increased neuronal activation in stress-sensitive brain nuclei and central regulation of salt and water balance area
			Variable increase in neuronal activation in central autonomic cell group
Andres-Hernando and colleagues [19]	Mice	Nephrectomy	Increased cytokine production and decreased renal clearance
Fuquay and colleagues [21]	Mice	IRI	Magnified humoral immune response
Liu and colleagues [25]	Mice	Nephrectomy, IRI	Increase of proinflammatory chemokines (keratinocyte-derived chemoattractant, monocyte chemoattractant protein-1, macrophage inflammatory protein) in the kidney
			Elevation of keratinocyte-derived chemoattractant, granulocyte colony-stimulating factor and glial fibrillary acidic protein in the brain
			No change in brain water content
			Increased blood–brain barrier permeability (extravasation of Evan blue dye in the brain)
Trachtman and colleagues [45]	Rats	Urethral ligation	Decreased brain water at 8 hours and increased organic osmolyte in the brain at 48 hours
Silver [47]	Rats	Urethral ligation	No significant change in brain organic osmolyte
			Decrease in brain water content
Galons and colleagues [48]	Rats	Nephrectomy	No difference in brain water content

AKI, acute kidney injury; IRI, ischaemic reperfusion injury.

How important these changes in neurotransmitters are in causing the alteration of cerebral function often witnessed in clinical practice is unknown, although the increased risk of developing delirium in the intensive care setting associated with the use of gamma aminobutyric acid_A_ agonists and anticholinergic drugs suggests that alterations in these neurotransmitters play a contributory role [32].

## Changes in endocrine function in acute kidney injury

Peptide hormones are freely filtered by the glomerulus and are then taken up by the proximal tubule, degraded and recycled as amino acids. However, most laboratories measure total hormone levels, a combination of free and protein-bound hormone, and some assays will measure both active hormone but also precursor and inactive hormones. As such, interpretation of hormone profiles in AKI has been difficult to disentangle. However, free T3 levels tend to fall in AKI, whereas catecholamines, vasopressin, natriuretic peptides and the renin–angiotensin–aldosterone axis increase. Renal sympathetic nervous system overactivity has been reported to increase the risk of hypertension and the development of the posterior reversible encephalopathy syndrome due to ischaemic injury and oedema in the medulla and cerebellum [33].

## Effect of inflammatory changes induced by acute kidney injury on the brain

Although the BBB regulates endothelial permeability, one should also remember that the endothelium-lining capillaries in the median eminence at the base of the third ventricle are fenestrated and as such will allow increased passage of cytokines and other inflammatory mediators into this area of the brain. Increasing circulating inflammatory cytokines (IL-1B, IL-8 and tumour necrosis factor alpha) in a rat model altered mRNA transcription proteins for nuclear factor-κB, cyclooxygenase-2, CCL2, CXCL1, IL-1B, and tumour necrosis factor alpha in the hypothalamic and hippocampus [34]. Furthermore, these changes were shown to lead to altered brain function, including decreased locomotor activity in a mouse model of AKI [25].

Interestingly, although this AKI model resulted in a significant increase in the generalised systemic inflammatory response with increased serum C-reactive protein, granulocyte colony-stimulating factor, IL-1B, IL-6 and keratinocyte-derived chemoattractant, there were significant differences in the inflammatory reaction within different organs, with increased macrophage inflammatory protein and monocyte chemoattractant protein-1 in the kidney, whereas in the cerebral cortex and corpus collosum of the brain there were substantial increases for keratinocyte-derived chemoattractant and granulocyte colony-stimulating factor, as well as glial fibrillary acidic protein, a specific cellular marker of brain inflammation [25] (Table 2). Importantly, glial fibrillary acidic protein did not increase in corresponding animal models of acute liver injury, suggesting that this effect was relatively specific for AKI and not just simply associated with generalised inflammation following other acute organ injury.

More recently, Ratliff and colleagues simplified the systemic inflammatory response in AKI in three waves of danger signalling, starting within minutes after renal ischaemia [18]. The first danger signal is associated with a surge in uric acid, a marker and mediator of renal ischaemic injury, triggering a secondary response, characterised by the exocytosis of Weibel–Palade bodies, releasing their proinflammatory mediators including endothelin-1, large multimers of von Willebrand factor, IL-8 and angiopoietin-2. Direct activation of the innate immune system via Toll-like receptors, as a result of the release of high mobility group box 1 protein and DNA binding protein, which act as potent cytokine-like mediators in the systemic circulation, leads to an intense inflammatory response within hours of ischaemic renal injury [18].

Synthesising the results of these studies, the inflammatory response observed in AKI leads to disruption of the BBB, endothelial injury and stimulation of the inflammatory and coagulation cascades within the brain via inflammatory cell responses leading to changes in neuronal cell protein transcription and cellular activation, altering cerebral function.

## Acid–base disturbances

The kidney plays a key role in acid–base balance regulation, to allow optimal cellular metabolism and function. AKI by predisposing to metabolic acidosis may play a detrimental role affecting cerebral neuronal metabolism and may impair normal cerebral function. One of the important enzyme systems in human cell metabolism is glutamate dehydrogenase, which uses ammonia, NADP(H) and NAD(H) to reversibly convert alpha-ketoglutarate to glutamate. Changes in acid–base balance might affect the affinity of protons for intermediary intracellular metabolism by altering energy generation. Increasing intracellular acidification leads to an increasing affinity of ammonia K_m_[NH4^+^] for human glutamate dehydrogenase, so resulting in oxidative deamination of glutamate [35], and thus altering neurotransmitter balance, with excess ammonia cycling between neurons and astrocytes [36].

In addition, in an increasingly intracellular acidic environment, protons can activate acid-sensing ion channels – resulting in an influx of both sodium and calcium into the cell, leading to cell membrane depolarisation and cellular injury and cell death [37,38]. Decreasing intracellular pH can induce calcium influx in an acid-sensing ion channels knockout cell model via voltage-gated calcium channels, resulting in an alteration of neural cell plasticity and increased cell injury [39].

Furthermore, the reduction in pH in the cerebrospinal fluid has local vasodilatory effects on the cerebral parenchymal arterioles through activation of large conductant, calcium-sensitive potassium channels [40], due to a combination of declining calcium wave activity and a significant elevation of calcium spark activity in the cerebral arteriole resulting in arteriolar vasodilatation with increased risk of localised cerebral oedema.

Metabolic acidosis also has other effects, including changing free concentrations to bound concentrations of calcium and magnesium, which impact on cellular ion fluxes, electrical gradients and neurotransmission, and also the protein binding of both azotaemic toxins and drugs that could affect their therapeutic effectiveness and also could result in accumulation and neurotoxicity [41].

## Organic osmolytes and brain water changes in acute kidney injury

AKI results in the retention of the waste products of nitrogen metabolism, typified by urea accumulation. Increased plasma urea leads to increased astrocyte and neuronal urea concentrations. Increasing intracellular urea risks intracellular hypertonicity and cell swelling in CKD. This outcome can be made worse by intracellular acidosis, which increases idiogenic osmoles. Diuretics may potentially exacerbate these effects by both reducing the effective plasma volume and cerebral perfusion, and also by causing electrolyte abnormalities [42]. Loop diuretics have been recently shown to alter aquaporin channel expression, so altering the passage of water through cells.

As the intracellular osmolality increases, glial cells and neurons initially compensate by excreting sodium, potassium and calcium and other osmotically active organic anions. However, depending upon the rate of urea production and when complicated by intracellular acidosis, the cellular compensatory mechanisms may become exhausted and glial cell and neuronal swelling occurs, with disruption of normal cellular metabolism, leading to uraemic encephalopathy described in CKD [43], which can produce a wide range of clinical manifestations from drowsiness to stupor, coma and even generalised seizures. In AKI, however, the time course of increasing serum urea and other azotaemic toxins is more rapid [44], and compensatory defence mechanisms take time to become fully operational. As such, the classic study of AKI in a rat model showed an accumulation of organic osmolytes in the brain tissues of rats within 48 hours of developing AKI, which was proceeded by a reduction of brain water at 8 hours [45]. This initial response is well recognised as a regulatory volume increase, with the brain transiently generating idiogenic osmoles derived from amino acids such as glutamine, taurine and inositol to equalise the osmolality gradient of hyperosmolal state in uraemia [46]. However, these findings have not been repeated by all investigators. For example, Silver did not find any difference in organic osmolytes, myoinositol, taurine, glutamate, glutamine, aspartate, alanine and glycine between uraemic nondialysed mice and nonuraemic controls using a mouse model, although significant decreases in brain water, measured by weight, were also observed in uraemic nondialysed mice when compared with normal control animals at 42 hours [47]. Other studies using different animal models failed to show any significant difference in the amount of brain water in nephrectomised nondialysed mice compared with normal control animals [25,48], and similarly for rat models of ischaemic AKI [29].

Unfortunately there are no comparative human studies in AKI; although Chen and colleagues showed significantly increased brain water measured by diffusion-weighted magnetic resonance imaging compared with normal controls [49], this study was performed in CKD patients, and as such the changes reported during chronic dialysis [50] may not be representative of those in AKI.

Although there are contradictory data between animal models in the accumulation of organic osmolytes and brain water, a regulatory brain volume increase would be expected to equalise the hyperosmolarity associated with AKI.

## Effect of acute kidney injury on organic ion transporters and drug metabolism

Organic anions and cations are excreted by the kidney by glomerular filtration and tubular secretion. Tubular secretion plays a major role in the excretion of not only organic acids but also exogenous chemical compounds. Organic anion transporters (OATs) are substrate specific for endogenous and exogenous anions [51]. OAT4 is located on the apical cell membrane for transporting anionic substrates from the luminal area into blood, whereas OAT1 to OAT3 are located on the basolateral membrane. Contrastingly, the organic cation transporters OCT1 to OCT3 are located on the basolateral membrane for transporting cationic compounds between the proximal tubules and blood. Some of these transporters are also found in the brain, with OCT3 and OAT3 expressed in the BBB and the BCSFB, modulating the efflux of organic solutes across the brain [52,53].

Indoxyl sulphate is an anionic and albumin-bound uremic toxin, and hence glomerular filtration is considered a minor process for excretion and tubular secretion is responsible for major excretion [54]. In rat models of CKD, OAT1 and OAT3 play a major role in transcellular transport for indoxyl sulphate [55] similar to humans [56]. Moreover, Schneider and colleagues reported decreased para-aminohippurate clearance, another protein-bound uraemic toxin, in an animal model of AKI associated with reduced mRNA expression for OAT1 and OAT3 [57], suggesting that OAT1 and OAT3 play a major role in uraemic toxin excretion in AKI. In uraemic animal models, downregulation of cerebral OAT3 plays a major role in reducing the efflux of drugs and organic solutes from the brain, which is exacerbated by competitive solute competition for the transporters [58,59].

Many sedatives, opioid analgesics and other drugs with central nervous actions have either significant parent drug or metabolite renal clearance, and as such have altered metabolism in AKI, with increased half-lives resulting in potential drug accumulation [59]. As such, the patient with AKI is at greater risk of developing a confused state. In addition, decreased OAT1 and OAT3 activity decreases methotrexate, nonsteroidal anti-inflammatory drug and acetylsalicylic acid elimination in AKI. Penicillin antibiotics compete for OAT transport, and thus patients with AKI are more at risk of developing penicillin-associated encephalopathy due to drug accumulation and increased brain concentrations. In addition to OATs, another family of cell membrane transporters – the ATP-dependent P-glycoprotein transporters – is also important in increasing drug clearance and is downregulated in AKI, potentially decreasing drug clearance and increasing drug exposure [60].

In addition, AKI not only reduces renal drug clearance but may also impair hepatic clearance by altering drug binding, on the one hand reducing protein binding of theophylline, phenytoin and methotrexate and on the other increasing protein binding of propranolol, cimetidine and clonidine [41]. Altering drug pharmacokinetics affecting the concentration of active metabolites may lead to drug accumulation and toxicity – for example, acyclovir and tacrolimus-induced encephalopathy – unless drug dosages are appropriately modified for renal function.

## Effect of acute brain injury on the kidney

Acute cerebral injury can lead to acute changes in renal sodium handling due both to changes in vasopressin secretion and to cerebral sodium wasting. Both excess anti-diuretic hormone section (syndrome of inappropriate antidiuretic hormone secretion) and cerebral sodium wasting may occur post acute subarachnoid haemorrhage and pituitary surgery, and although both cause hyponatraemia, clinical examination should establish that patients with syndrome of inappropriate antidiuretic hormone secretion are euvolaemic or hypervolaemic, whereas patients with cerebral salt wasting are typically hypovolaemic (Table 3) [61].

**Table 3:** Differences and similarities between cerebral sodium wasting and syndrome of inappropriate antidiuretic hormone

	**Cerebral sodium wasting**	**Syndrome of inappropriate antidiuretic hormone**
Extracellular volume	↓	Normal or ↑
Urine sodium concentration	Normal or ↑	Normal or ↑
Plasma renin	± ↑	± ↓
Plasma aldosterone	↑	± ↓
Serum urate	↓↓	↓ or normal
Fractional excretion urate	↑↑	↑ or normal
Fractional excretion phosphate	± ↑	Normal

↓, decreased; ↑, increased; ↓↓, greatly decreased; ↑↑, greatly increased.

Acute cerebral injury is often associated with increased visceral sympathetic nervous system activity and plasma catecholamines resulting in systolic hypertension. Although this may be a compensatory mechanism to preserve perfusion to areas of damaged brain, increased visceral sympathetic nervous system activation results in reduced renal glomerular perfusion with increased renal sodium reabsorption. Sustained severe hypertension may lead to red cell fragmentation, haemolysis and AKI secondary to red cell thrombi in the glomeruli, similar to that found with thrombotic thrombocytopenic purpura.

## Acute irreversible brain injury

The increasing use of cadaveric kidneys from brain-dead donors has led to research into the effects of brain death on renal function. Sanchez-Fructuoso and colleagues reported that the renal allograft survival rate from deceased donors was significantly lower than that for living related donors [62]. Furthermore, other studies reported that donor brain death was correlated with both delayed allograft function and increased acute rejection [63]. Brain death may affect the kidney by a combination of haemodynamic effects [64], neurohormonal activation [65], inflammatory response [66] and endothelial activation [67]. Most of the recent studies have concentrated on the inflammatory response in the allograft, with reports of both increased upregulation of kidney injury molecule-1 and inflammatory cytokines including IL-8 and IL-10, but also increased numbers of trafficking inflammatory cells such as FoxP3^+^ regulatory T cells [68], leading to reduce allograft survival [5].

## Effect of acute kidney injury on outcomes in patients with traumatic brain injury, secondary to subarachnoid haemorrhage and post cardiac arrest

The majority of patients with acute traumatic brain injury are young and have normal pre-existing renal function, but even so the reported incidence of AKI stage 1 has been reported to be around 23%, with only a small minority requiring renal replacement therapy. However, the mortality in some studies increased fivefold for patients with AKI [69]. In other studies, greater use of mannitol was associated with an increased incidence of AKI, and intracranial pressure monitoring may reduce the risk of AKI by reducing mannitol usage [70].

Although patients with acute subarachnoid haemorrhage are usually older with a past history of hypertension, the incidence of AKI has also been reported to be around 23%, but with a twofold increase in worse functional recoveries and the mortality increasing with severity of AKI class [71]. Somewhat surprisingly, as patients suffering cardiac arrest may be expected to have some underlying degree of pre-existing cardiac and vascular disease, the rates of AKI reported have been lower – 18% AKI stage 1 and 10% AKI stage 3 – with no reported excess mortality with AKI [72].

## Management of acute kidney injury in the patient with acute brain injury

The majority of patients with acute traumatic brain injury are young and have normal renal function. However, they may be at risk of AKI secondary to blood loss from multiple trauma, exposure to radiocontrast agents and prescription of nephrotoxic antibiotics to treat sepsis and nonsteroidals for analgesia. On the other hand, patients admitted with subarachnoid haemorrhage and acute stroke are usually older and more likely to have a background of CKD secondary to hypertension and small vessel disease associated with diabetes and cardiovascular disease. As such, maintaining adequate hydration is an important clinical goal for preventing AKI, particularly for reducing the risk of contrast-induced nephropathy. In addition, multiple contrast exposure should be minimised by selecting other imaging techniques wherever possible, and nephrotoxic drugs should be similarly avoided if possible.

## Renal replacement therapy for acute kidney injury in patients with acute brain injury

Although renal replacement therapy can help improve encephalopathy due to uraemia, by removing azotaemic retention products and reducing toxic drug levels, too rapid a fall in serum urea may paradoxically cause cerebral oedema.

Intermittent haemodialysis using modern-day high-efficiency dialysers coupled with dialysates containing supraphysiological levels of bicarbonate can exacerbate underlying brain damage. Urea is rapidly removed from the plasma water during intermittent haemodialysis, but there is a delay in urea moving out of cells, including neurons and cerebrospinal fluid [73]. This leads to the development of a concentration gradient, and – as water movement through aquaporins is some 20 times faster than urea transport – water will move along the concentration gradient generated by the difference in urea concentrations, passing into the brain, leading to brain swelling [74]. Similarly the current practice, particularly in the United States, of choosing dialysate bicarbonate concentrations ≥38 mmol/l poses a problem, because under normal conditions cerebrospinal pH is a little lower than that of blood, and brain intracellular pH lower still. The rapid increase in blood pH during dialysis sets up a disequilibrium; as bicarbonate is charged it only slowly crosses into cells, whereas the reaction between bicarbonate and hydrogen ions in plasma water leads to carbon dioxide, which rapidly transverses lipid-rich cell membranes and once inside cells generates hydrogen ions, generating a paradoxical intracellular acidosis, which itself leads to generation of intracellular idiogenic osmoles, so increasing osmolality further and increasing water entry. In addition, these changes in cerebral pH adversely affect the respiratory centre in those patients spontaneously breathing, increasing the risk and frequency of apneic spells. As such, continuous forms of renal replacement therapy are to be preferred so that the rate of change in serum urea and bicarbonate are slower, and risk of brain swelling is reduced [75,76].

It is equally important to avoid intradialytic hypotension to minimise iatrogenic episodes of cerebral ischaemia [77]. The risk of cardiovascular stability during dialysis can be reduced by cooling the dialysate to 35°C, and increasing the dialysate sodium to around 10 mEq/l above serum sodium [78]. As such, slowing down the intermittent haemodialysis session by moving towards prolonged intermittent renal replacement therapy, with slower blood and dialysate flows coupled with high dialysate sodium and cooling, as can be achieved with the Genius® (Fresenius AG, BadHomburg, Germany) dialysis system, may prove equally as effective as continuous renal replacement therapy in preventing an increase in cerebral oedema. However, prolonged intermittent renal replacement therapy using a combination of higher blood flows, warmed and lower sodium hypotonic dialysates risks an increase in intracranial pressure [79,80]. Peritoneal dialysis is a continuous renal replacement therapy and, although associated with slower solute clearances than haemodialysis, can also lead to an increase in brain swelling in brain-injured patients, as all commercial dialysates are hyponatraemic and risk exacerbating hyperglycaemia due to the high glucose content. Also, instillation and drainage of hypertonic glucose dialysates can lead to swings in right atrial filling, with a consequent reduction in cardiac output that may compromise cerebral perfusion [76,81].

## Conclusion

Cerebral dysfunction is a common finding in patients with AKI. Although alterations of mental status are more likely to occur in the older patient with pre-existing small vessel cerebrovascular disease, sepsis is probably the most common triggering factor. However, AKI is an inflammatory state *per se*, affecting distant organ injury mediated by increased circulating cytokines and other inflammatory mediators, due to a combination of increased production and reduced clearance. Increased circulating cytokines and other inflammatory mediators predispose to alterations in the BBB, leading to increased permeability and changes in neuronal homeostasis. This is exacerbated by changes in excitatory and inhibitory neurotransmitter concentrations. AKI leads to the retention of azotaemic products of nitrogen metabolism and metabolic acidosis, which lead to changes in intracellular osmolality and further disturb intracellular homeostasis. Alterations in drug pharmacokinetics in patients with AKI may then lead to parent drug or metabolite accumulation, which may further alter higher mental functions and increase the risk for cerebral dysregulation.

Patients admitted with acute cerebral dysfunction are at risk of AKI primarily secondary to hypovolaemia, reduced intrarenal glomerular perfusion, and exposure to radiocontrast and nephrotoxic drugs. As such, achieving adequate hydration and avoiding or minimising nephrotoxin exposure is key to preventing AKI. For patients who develop progressive AKI requiring renal replacement therapy, modifying the dialysis prescription may minimise further brain injury. These modifications would include continuous renal replacement therapy or intermittent haemodialysis with reduced blood flow rate, a longer daily dialysis session time in combination with smaller surface area biocompatible dialysers, and cooled dialysate with increased sodium and reduced bicarbonate dialysate concentrations.

## Abbreviations

AKI: Acute kidney injury; BBB: Blood–brain barrier; BCSFB: Blood–cerebrospinal fluid barrier; CKD: Chronic kidney disease; IL: Interleukin; OAT: Organic anion transporter; OCT: Organic cation transporter.

## Competing interests

The authors declare that they have no competing interests.
